# New spectrofluorimetric methods for determination of melatonin in the presence of *N*-{2-[1-({3-[2-(acetylamino)ethyl]-5-methoxy-1*H*-indol-2-yl}methyl)-5-methoxy-1*H*-indol-3-yl]- ethyl}acetamide: a contaminant in commercial melatonin preparations

**DOI:** 10.1186/1752-153X-6-36

**Published:** 2012-05-02

**Authors:** Hany W. Darwish, Mohamed I. Attia, Darius P. Zlotos

**Affiliations:** 1 Department of Pharmaceutical Chemistry, College of Pharmacy, King Saud University, P.O. Box 2457, Riyadh, 11451 Saudi Arabia; 2 Department of Analytical Chemistry, Faculty of Pharmacy, Cairo University, Kasr El-Aini Street, ET 11562 Cairo, Egypt; 3 Department of Pharmaceutical Chemistry, Institute of Pharmacy and Food Chemistry, Würzburg University, Am Hubland, 97074 Würzburg, Germany; 4 Department of Pharmaceutical Chemistry, Faculty of Pharmacy and Biotechnology, The German University in Cairo, New Cairo City, Egypt

**Keywords:** Melatonin, Contaminants, Synthesis, Spectrofluorimetry, Commercial preparations

## Abstract

**Background:**

Melatonin (MLT) has many health implications, therefore it is of valuable importance to develop specific analytical methods for determination of MLT in the presence of its main contaminant, *N*-{2-[1-({3-[2-(acetylamino)ethyl]-5-methoxy-1*H*-indol-2-yl}methyl)-5-methoxy-1*H*-indol-3-yl]ethyl}acetamide (**10**). For development of these analytical methods, compound **10** had to be prepared in an adequate amount.

**Results:**

Compound **10** was synthesized in six steps starting from 5-methoxyindole-2-carboxylic acid (**1**). Analytical performance of the proposed spectrofluorimetric methods was statistically validated with respect to linearity, accuracy, precision and specificity. The proposed methods were successfully applied for the assay of MLT in laboratory prepared mixtures containing up to 60 % of compound **10** and in commercial MLT tablets with recoveries not less than 99.00 %. No interference was observed from common pharmaceutical additives and the results were favorably compared with those obtained by a reference method.

**Conclusions:**

This work describes simple, sensitive, and reliable second derivative spectrofluorimetric method in addition to two multivariate calibration methods**,** principal component regression (PCR) and partial least square (PLS)**,** for the determination of MLT in the presence of compound **10**.

## Background

**Figure 1:**
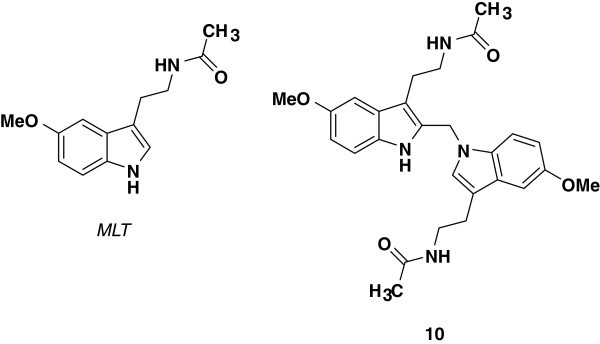
**Structures of melatonin (MLT) and compound 10.**

Melatonin (*N*-acetyl-5-methoxytryptamine, MLT, Figure 1) is primarily produced by the pineal gland in the brain with a marked circadian rhythm normally peaking in the dark to regulate sleep. MLT acts through activation of two G-protein-coupled receptors, designated as MT_1_ and MT_2_[1]. In addition, a low-affinity putative MLT binding site called MT_3_ has been recently characterized as a melatonin-sensitive form of the human enzyme quinine reductase 2 [2]. MLT has found widespread use in the treatment of sleep disorders, other effects described in the literature include its anti- inflammatory, pain modulatory, antitumor, and antioxidant properties [3–8].

MLT as well as L-tryptophane (Trp) are naturally occurring indole-based compounds and are sold over-the-counter as dietary supplement in the United States. Contaminants in Trp preparations are the etiological agents for the 1989 outbreak of oesinophilia-myalgia syndrome (EMS), which affected about 1500 people and led to about 30 deaths in the United States [9]. Administration of 15 mg/day MLT for four weeks to cancer patients has led to induction of oesinophilia [10, 11]. Accordingly, Williamson *et al.*[12] investigated the presence of contaminants structurally related to those found in contaminated Trp preparations. They reported the presence of six structural analogues of Trp contaminants in three different commercial MLT preparations. Two contaminants were identified to be hydroxymelatonin isomers with MH^+^ = 249 whereas, other four contaminants were identified as melatonin-formaldehyde condensation products with MH^+^ = 477. Consequently, a tighter control on nutritional supplements sold and used as drugs was recommended.

Compound **10** (Figure 1) is the most abundant regio-isomer from the four melatonin-formaldehyde condensation contaminants that were found in the commercial preparations of MLT [12]. To the best of our knowledge, a preparative methodology for the synthesis of pure **10** has not reported yet. We therefore report herein a preparative method for the synthesis of compound **10**, which is required for the development of spectrofluorimetric methods for determination of MLT in the presence of compound **10** in commercial MLT preparations. An evaluation of the literature revealed that only one HPLC/tandem mass spectrometry (LC/MS/MS) method has been published for the determination of MLT in the presence of compound **10**[12]. This method offers a high degree of specificity, however its sophisticated instrumentation and cost factor preclude its use in routine analysis. Therefore, it was desirable to develop simple and fast procedures that could be applied in quality control laboratories for the determination of MLT in presence of compound **10**. Derivative spectrofluorimetry and multivariate calibration methods such as PCR and PLS are useful means of resolving two overlapping spectra and eliminating matrix interference in the assay of two-component mixtures [13, 14].

The principal advantages of these methods lie in the improved sensitivity and selectivity, in addition to the significant economic advantages over other sophisticated instrumental techniques such as HPLC/tandem mass spectrometry.

## Experimental

Melting points were determined using a capillary melting point apparatus (Gallenkamp, Sanyo) and are uncorrected. Column chromatography was carried out on silica gel 60 (0.063–0.200 mm) obtained from Merck. A Bruker AV-400 spectrometer was used to obtain ^1^ H NMR (400 MHz) and ^13^ C NMR (100 MHz) spectra. The NMR resonances were assigned by means of HH-COSY, HMQC, and HMBC experiments. EI mass spectra were determined on a Finnigan MAT 8200 spectrometer. IR spectra, recorded as ATR, were obtained by using a Biorad PharmalyzIR FT-IR instrument. Fluorescence measurements were carried out using a Shimadzu (Kyoto, Japan) RF-5301 version 3.0 spectrofluorimeter equipped with a 150 W xenon lamp and 1 cm quartz cells. The slit width of both the excitation and emission monochromators was set at 5 nm. The calibration and linearity of the instrument were frequently checked with standard quinine sulphate (0.01 μg ml^-1^). Wavelength calibration was performed by measuring λ_excitation_ at 279 nm and λ_emission_ at 333 nm; no variation in the wavelength was observed. Elemental analyses were performed by the microanalytical section of the Institute of Inorganic Chemistry, University of Würzburg. All reactions were carried out under an argon atmosphere. MLT was obtained from Merc Inc, New York, USA. Its purity is certified to be 99.5%. Methanol and ethyl acetate were of analytical-reagent grade. Commercially available MLT preparation labeled to contain 3 mg MLT was purchased from the local market.

## Synthesis

### (5-Methoxy-2,3-dihydro-1 *H*-indol-1-yl)(5-methoxy-1 *H*-indol-2-yl)methanone (3)

A solution of 5-methoxyindoline (**2**) (0.94 g, 6.28 mmol) in dry CH_2_Cl_2_ (5 ml) was added to a stirred solution of 5-methoxyindole-2-carboxylic acid (**1**) (1.2 g, 6.28 mmol) and ethyl-3-(3-dimethylaminopropyl)carbodiimide hydrochloride (EDCI·HCl) (1.80 g, 9.42 mmol) in dry CH_2_Cl_2_ (15 ml). The reaction mixture was stirred for 18 h at room temperature, extracted with 5 N hydrochloric acid (3 × 5 ml), washed with water (2 × 10 ml), and dried (Na_2_SO_4_). The organic layer was evaporated in vacuo, and the residue was recrystallized from isopropanol to yield 1.76 g (87%) of **3** (1.76 g, 87%) as a pale yellow powder mp 234–236°C. FTIR (ATR) ν = 3270, 2935, 1604, 1576, 1406, 797 cm^-1^. ^1^ H NMR (DMSO-*d*_*6*_): δ 3.26 (t, 2 H, *J* = 8.4 Hz, H-3′), 3.79 (s, 3 H, OCH_3_), 3.81 (s, 3 H, OCH_3_), 4.52 (t, 2 H, *J* = 8.4 Hz, H-2´), 6.83 (dd, 1 H, *J* = 8.8, 2.5 Hz, H-6), 6.93 (d, 1 H, *J* = 2.5 Hz, H-4′), 6.95-6.96 (m, 1 H, H-6′), 7.04 (s, 1 H, H-3), 7.15 (d, 1 H, *J* = 2.5 Hz, H-4), 7.43 (d, 1 H, *J* = 8.8 Hz, H-7), 8.14 (d, 1 H, *J* = 8.6 Hz, H-7′), 11.58 (br., 1 H, NH). ^13^ C NMR (DMSO-*d*_6_): δ 28.4 (C-3′), 49.7 (C-2′), 55.3 (OCH_3_), 55.4 (OCH_3_), 102.1 (C-4), 104.9 (C-3), 110.7 (C-6′), 111.9 (C-6), 113.1 (C-7), 115.1 (C-4′), 117.6 (C-7′), 127.6, 131.2, 131.4, 133.9, 136.9 (ArC), 153.8, 156.1 (C-5, C-5′), 159.5 (O = C). MS (EI): *m/z* (%) = 322 (M^+^, 27), 174 (10), 149 (100), 134 (29). Anal. Calcd for C_19_H_18_N_2_O_3_: C, 70.79; H, 5.63; N, 8.69. Found: C, 70.41; H, 5.61; N, 8.75.

### (5-Methoxy-1 *H*-indol-1-yl)(5-methoxy-1 *H*-indol-2-yl)methanone (4)

A mixture of **3** (0.20 g, 0.62 mmol) and 2,3-dichloro-5,6-dicyanobenzoquinone (DDQ) (0.19 g, 0.68 mmol) was heated at reflux temperature in ethyl acetate (30 ml) for 18 h. The reaction mixture was evaporated under reduced pressure and the residue was purified by silica gel chromatography (chloroform/methanol/ammonia, 10:1:0.1) to furnish 0.19 g (96%) of **4** as a light red powder mp 178–179°C and. FTIR (ATR) ν = 3290, 2937, 1625, 1517, 1026, 797 cm^-1^.^1^ H NMR (CDCl_3_): δ 3.85 (s, 3 H, OCH_3_), 3.87 (s, 3 H, OCH_3_), 6.63 (d, 1 H, *J* = 3.8 Hz, H-3′), 6.99 (dd, 1 H, *J* = 9.1, 2.5 Hz, H-6′), 7.03 (dd, 1 H, *J* = 8.8, 2.2 Hz,, 7.07 (d, 1 H, *J* = 2.2 Hz, H-4), 7.08-7.09 (m, 2 H, H-3, H-4′), 7.37 (d, 1 H, *J* = 8.8 Hz, H-7), 7.90 (d, 1 H, *J* = 3.8 Hz, H-2′), 8.37 (d, 1 H, *J* = 9.1 Hz, H-7′), 9.70 (br., 1 H, NH). ^13^ C NMR (CDCl_3_): δ 55.7 (2 x OCH_3_), 102.4 (C-4), 103.7 (C-3′), 108.9 (C-3), 109.6 (C-7), 113.0 (C-6′), 113.4 (C-6), 116.9 (C-7′), 117.7 (C-4′), 127.6 (C-2′), 127.8, 129.2, 130.8, 131.6, 132.5 (ArC), 154.9, 156.7 (C-5, C-5′), 160.8 (O = C). MS (EI): *m/z* (%) = 320 (M^+^, 70), 173 (53), 147 (100), 119 (29). Anal. Calcd for C_19_H_16_N_2_O_3_: C, 71.24; H, 5.03; N, 8.75. Found: C, 70.95; H, 5.08; N, 8.68.

### 5-Methoxy-1-[(5-methoxy-1 *H*-indol-2-yl)methyl]-1 *H*-indole (5)

Compound **4** (0.50 g, 156.03 mmol) was dissolved in dry THF (5 ml) and was added dropwise to a cooled (0°C) suspension of LiAlH_4_/AlCl_3_ in dry diethyl ether (prepared by a slow addition of AlCl_3_ (0.32 g, 2.41 mmol) to a suspension LiAlH_4_ (0.27 g, 7.13 mmol) in dry diethyl ether (15 ml) at 0°C. The resulting reaction mixture was stirred at 0°C for one hour and at room temperature for another one hour. The reaction was quenched by a slow addition of saturated sodium sulphate solution. The solids were removed by filtration, washed with chloroform (20 ml) and the combined organic phase was dried (Na_2_SO_4_) and evaporated under reduced pressure. The residue was purified by silica gel chromatography (chloroform/methanol/ammonia, 10:1:0.1) to produce 0.4 g (83%) of **5** as a light red powder mp 173–174°C. FTIR (ATR) ν = 3384, 2956, 1622, 1485, 795 cm^-1^.^1^ H NMR (CDCl_3_): δ 3.83 (s, 3 H, OCH_3_), 3.85 (s, 3 H, OCH_3_), 5.26 (s, 2 H, CH_2_-N), 6.39 (s, 1 H, H-3) 6.49 (d, 1 H, *J* = 3.3 Hz, H-3′), 6.81 (dd, 1 H, *J* = 8.8, 2.3 Hz, H-6′), 6.86 (dd, 1 H, *J* = 8.8, 2.5 Hz, H-6), 7.01 (d, 1 H, *J* = 8.8 Hz, H-7′), 7.04-7.05 (m, 2 H, H-2′, H-4), 7.15 (d, 1 H, *J* = 2.3 Hz, H-4′), 7.20 (d, 1 H, *J* = 8.8 Hz, H-7), 7.74 (br., 1 H, NH). ^13^ C NMR (CDCl_3_): δ 44.0 (CH_2_-N), 55.8 (OCH_3_), 55.9 (OCH_3_), 101.3 (C-3), 101.6 (C-3′), 102.3 (C-4), 102.8 (C-4′), 110.1 (C-6′), 111.6 (C-6), 112.2, 112.3 (C-7, C-7′), 128.4, 128.5 (ArC), 129.2 (C-2′), 131.4, 131.6, 134.7 (ArC), 154.2 (C-5, C-5′). MS (EI): *m/z* (%) = 306 (M^+^, 35), 160 (100), 147 (23). Anal. Calcd for C_19_H_18_N_2_O_2_: C, 74.49; H, 5.92; N, 9.14. Found: C, 74.19; H, 5.95; N, 8.89.

### 2-{[5-Methoxy-3-(2-nitroethyl)-1 *H*-indol-1-yl]methyl}-5-methoxy-3-(2-nitroethyl)-1 *H*-indole (8)

A mixture of **5** (0.250 g, 0.82 mmol), 2-nitroethyl acetate (0.350 g, 2.63 mmol), and *tert*. butyl catechol (6 mg) in xylene (20 ml) was heated at reflux temperature for 18 h. The reaction mixture was evaporated under vacuum and the residue was purified by silica gel chromatography (chloroform/methanol, 9:0.5) to yield 0.17 g (46%) of **8** as brown viscous oil. FTIR (ATR) ν = 3421, 2965, 1548, 1212, 795 cm^-1^. ^1^ H NMR (CDCl_3_): δ 3.39 (t, 2 H, *J* = 7.1 Hz, C*H*_2_-CH_2_-N), 3.44 (t, 2 H, *J* = 6.8 Hz, C*H*_2_-CH_2_-N), 3.83 (s, 3 H, OCH_3_), 3.84 (s, 3 H, OCH_3_), 4.56 (t, 2 H, *J* = 6.8 Hz, CH_2_-C*H*_2_-N), 4.61 (t, 2 H, *J* = 7.1 Hz, CH_2_-C*H*_2_-N), 5.30 (s, 2 H, CH_2_-N), 6.81 (dd, 1 H, *J* = 8.8, 2.3 Hz, ArH), 6.86 (dd, 1 H, *J* = 8.8, 2.5 Hz, ArH), 6.88 (s, 1 H, H-2′), 6.80 (d, 1 H, *J* = 2.4 Hz, ArH), 6.98 (d, 1 H, *J* = 2.4 Hz, ArH), 7.09 (d, 1 H, *J* = 8.8 Hz, ArH), 7.14 (d, 1 H, *J* = 8.8 Hz, ArH), 7.66 (br., 1 H, NH). ^13^ C NMR (CDCl_3_): δ 22.6 (*C*H_2_-CH_2_-N), 23.5 (*C*H_2_-CH_2_-N), 42.0 (CH_2_-N), 55.9 (OCH_3_), 56.0 (OCH_3_), 74.9 (CH_2_-*C*H_2_-N), 75.7 (CH_2_-*C*H_2_-N), 100.0, 100.6, 110.4, 112.1, 112.6, 112.8 (ArCH), 126.7 (C-2′), 109.5 (C-3, C-3′), 128.9, 130.6, 131.9, 132.0, 133.4 (ArC), 154.5, 154.6 (C-5, C-5′). MS (EI): *m/z* (%) = 452 (M^+^, 17), 233 (58), 186 (100). Anal. Calcd for C_23_H_24_N_4_O_6_: C, 61.06; H, 5.35; N, 12.38. Found: C, 60.86; H, 5.24; N, 12.49.

### *N*-{2-[1-({3-[2-(Acetylamino)ethyl]-5-methoxy-1 *H*-indol-2-yl}methyl)-5-methoxy-1 *H*-indol-3-yl]ethyl}acetamide (10)

A mixture of **8** (0.17 g, 0.38 mmol) and 10% Pd/C (70 mg) in absolute ethanol (10 ml) was hydrogenated under 4 mbar pressure in Parr shaker device at ambient temperature for 18 h. The reaction mixture was filtered off and the filtrate was evaporated under reduced pressure to furnish 0.15 g of **9** as pale yellow viscous oil. Crude **9** (0.15 g, 0.38 mmol) was acetylated using acetic anhydride (0.36 ml, 3.82 mmol) and triethylamine (0.38 ml, 2.66 mmol) in dry DCM (10 ml) at room temperature for 18 h. The solvent was evaporated under vacuum and the residue was purified by silica gel chromatography (chloroform/methanol/ammonia, 10:1:0.1) to yield 0.11 g (63%) of **10** as a beige powder mp 88–90°C and was FTIR (ATR) ν = 3286, 2924, 1635, 1216, 794 cm^-1^.^1^ H NMR (CDCl_3_): δ 1.76 (s, 3 H, CH_3_), 1.80 (s, 3 H, CH_3_), 2.84 (t, 2 H, *J* = 6.6 Hz, C*H*_2_-CH_2_-N), 2.96 (t, 2 H, *J* = 6.8 Hz, C*H*_2_-CH_2_-N), 3.39-3.44 (m, 2 H, CH_2_-C*H*_2_-N), 3.48-3.53 (m, 2 H, CH_2_-C*H*_2_-N), 3.81 (s, 3 H, OCH_3_), 3.82 (s, 3 H, OCH_3_), 5.25 (s, 2 H, CH_2_-N), 5.74 (t, 1 H, *J* = 5.7 Hz, NH), 5.83 (t, 1 H, *J* = 5.6 Hz, NH), 6.78 (dd, 1 H, *J* = 8.8, 2.3 Hz, ArH), 6.81 (dd, 1 H, *J* = 8.8, 2.5 Hz, ArH), 6.87 (s, 1 H, H-2′), 6.99 (d, 2 H, *J* = 2.3 Hz, ArH), 7.11 (d, 1 H, *J* = 8.8 Hz, ArH), 7.14 (d, 1 H, *J* = 8.8 Hz, ArH), 8.24 (br., 1 H, NH). ^13^ C NMR (CDCl_3_): δ 23.1 (CH_3_), 23.2 (CH_3_), 24.3 (*C*H_2_-CH_2_-N), 25.3 (*C*H_2_-CH_2_-N), 39.8 (CH_2_-*C*H_2_-N), 40.2 (CH_2_-*C*H_2_-N), 41.9 (CH_2_-N), 55.9 (2 x OCH_3_), 100.5, 100.9, (ArCH), 110.1, 110.3 (C-3, C-3′), 111.9, 112.3, 112.4, 112.5 (ArCH), 126.2 (C-2′), 128.5, 128.8, 130.9, 131.4, 131.9 (ArC), 154.1, 154.2 (C-5, C-5′), 170.3 (O = C), 170.5 (O = C). MS (EI): *m/z* (%) = 476 (M^+^, 31), 417 (16), 245 (100), 203 (41), 186 (64). Anal. Calcd for C_27_H_32_N_4_O_4_: C, 68.05; H, 6.77; N, 11.76. Found: C, 68.37; H, 6.59; N, 11.66.

## Analysis

### Preparation of MLT and compound 10 standard solutions

Stock solutions of MLT (100 μg ml^-1^) and compound **10** (300 μg ml^-1^) were prepared by dissolving 10 mg and 30 mg of MLT and compound **10**, respectively, in 100 ml methanol. Appropriate volumes of these stock solutions were diluted to give working solutions of 4 and 3 μg ml^-1^for MLT and compound **10**, respectively. Stock and working solutions were stable for at least two weeks when stored refrigerated at 4°C.

### Preparation of MLT tablets sample solutions

Ten tablets were weighed and finely powdered. An accurately weighed portion of the powder equivalent to 3 mg of MLT was extracted with ethyl acetate and the extract was filtered. The extract was evaporated and reconstituted in methanol to obtain final concentration of 4 μg ml^-1^ MLT. Aliquots of tablet extract were diluted with methanol to obtain final concentration of 120 ng ml^-1^ and the samples were subjected to the analysis according to the **Calibration procedures**.

### Calibration procedures

#### Second derivative method

Aliquots equivalent to 20–220 ng ml^-1^ MLT were accurately transferred from its standard working solution into separate series of 5-ml volumetric flasks then completed to volume with methanol. The emission spectra of the prepared standard solutions were scanned from 300 to 450 nm using λ_excitation_ at 279 nm and stored in the computer. The second derivative of stored emission spectra of MLT were computed with Δ*λ* = 10 nm. The amplitude of the second derivative peak of MLT was measured at 324.0 nm. The calibration graph was constructed by relating the peak amplitudes at 324.0 nm to the corresponding concentrations of MLT and the regression equation for the data was computed.

#### PCR and PLS chemometric models

Four level, two factor calibration design [15] was used for construction of 16 samples of the calibration set by transferring different volumes of MLT and compound **10** from their standard working solutions into 5-ml volumetric flasks then completed to volume with methanol (Table 1).

**Table 1:** **The four level two factor experimental design of the calibration set mixtures shown as concentrations of the mixture components in ng ml** ^**-1**^

Mix. No.	MLT	Compound 10	Mix. No.	MLT	Compound 10
1	40	10	9	100	10
2	40	20	10	100	20
3	40	40	11	100	40
4	40	50	12	100	50
5	60	10	13	120	10
6	60	20	14	120	20
7	60	40	15	120	40
8	60	50	16	120	50

The emission spectra of the calibration set were scanned from 300 to 380 nm using λ_excitation_ at 279 nm and stored in the computer. Mean centering of the data proved to be the best preprocessing method for getting the optimum results.

##### Constructing the PCR and PLS models

The calibration set fluorescence intensities and their corresponding concentrations were used to build the PCR and PLS models using PLS-Toolbox 2.0 software for the calculations.

##### Selection of the optimum number of factors to build the PCR and PLS models

The cross validation method, leaving out one sample at a time, was used to select the optimum number of factors [16]. Given a set of 16 calibration samples, the PCR and PLS calibrations were performed on 15 samples. By using this calibration, the concentration of the sample left out was predicted. This process was repeated a total of 16 times until each sample had been left out once. The predicted concentrations were then compared with the known concentrations. The root mean square error of cross validation (RMSECV) was calculated in the same manner each time a new factor was added to the model. The maximum number of factors used to calculate the optimum RMSECV was selected to be nine. The method described by Haland and Thomas [17] was used for selecting the optimum number of factors.

### Assay of laboratory prepared mixtures

Aliquots of MLT and compound **10** were transferred from their standard working solutions into a series of 5-ml measuring flasks, completed to volume with methanol and mixed well. For determination of MLT, by the proposed methods, the **Calibration procedures** were applied.

## Results and discussion

MLT dietary supplement tablets are mainly used in treatment of sleep disorders and are sold over-the-counter. It was reported that commercial MLT preparations contain six contaminants; four of them (MLT-formaldehyde condensation contaminants) are thought to be responsible for induction of oesinophilia when MLT was administered in a high dose for long time [10–12]. Compound **10** is the most abundant regio-isomer from these four formaldehyde condensation contaminants [12]. Daily value of MLT intake is not established yet and therefore, it is of sizable importance to develop simple, accurate, and sensitive methods for the routine analysis of MLT in the presence of its main contaminant, compound **10**.

**Scheme 1:**
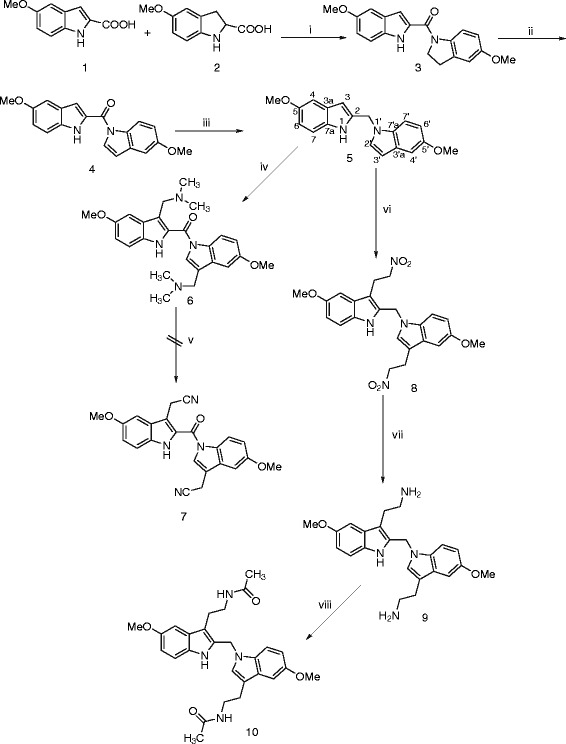
**Synthetic pathway for preparation of compound 10.** Reagents and conditions: i) EDCI.HCl, DCM, rt, 18h; ii) DDQ, ethyl acetate, reflux, 18h; iii) LiAlH_4_/AlCl_3_, THF/Et_2_O, 0°C-rt, 2h; iv) dimethyl amine, HCHO, CH_3_COOH; v) 1. MeI, CH_2_CL_2_, 2. KCN, dicyclohexyl[18]-crown[6], MeCN; vi) 2-nitroethyl acetate, *tert*. butyl catechol, xylene, reflux, 18h; vii) H_2_, Pd/C, 4 mbar, rt, 18h; viii) acetic anhydride, Et_3_N, DCM, rt, 18h.

To develop such analytical methods, a considerable amount of pure compound **10** was required to be prepared. Synthesis of the target compound **10** was carried out according to the synthetic pathway depicted in **Scheme** 1. Thus, the commercially available 5-methoxyindole-2-carboxylic acid (**1**) was allowed to react with the nucleophile 5-methoxyindoline (**2**) [18] in the presence of coupling reagent ethyl-3-(3-dimethylaminopropyl)-carbodiimide hydrochloride (EDCI.HCl) in DCM at room temperature to furnish the amide **3** in good yield. Subsequent oxidation of indoline ring of **3** was accomplished using 2,3-dichloro-5,6-dicyanobenzoquinone (DDQ) in ethyl acetate [19] at reflux temperature to yield the di-indole derivative **4**. Trials to reduce amide bond in **4** using LiAlH_4_ in THF or in diethyl ether led to cleavage of the amide bond. Reduction of the amide bond in compound **4** was successfully achieved using LiAlH_4_/AlCl_3_ (3/1) mixture in THF/diethyl ether solvent system at 0°C for one hour and then at room temperature for another one hour. Introduction of the aminoethyl side chains into positions 3 and 3′ of compound **5** *via* adopting our previously reported procedure [20] was unsuccessful. Briefly, compound **5** was subjected to Mannich reaction using dimethylamine and formaldehyde in glacial acetic acid produced the Mannich base **6**. Subsequent quaternization of **6** with methyl iodide followed by substitution with potassium cyanide in the presence of dicyclohexyl[18]-crown[6] did not yield the anticipated compound **7** which might be reduced to its respective diamine derivative that could produce the target compound **10** upon acetylation. Accordingly, another strategy was adopted to synthesize **10**. Thus, 2-nitroethyl acetate [21] was reacted with **5** in xylene at reflux temperature to yield the di-nitro derivative **8** which was catalytically hydrogenated in Parr shaker device at 4 mbar pressure to furnish compound **9**. Acetylation of **9** using acetic anhydride and triethylamine in DCM produced the target compound **10**. Assigned structures of the synthesized compounds were characterized by ^1^ H NMR, ^13^ C NMR, and MS spectral data whereas, purity was determined *via* microanalyses.

**Figure 2:**
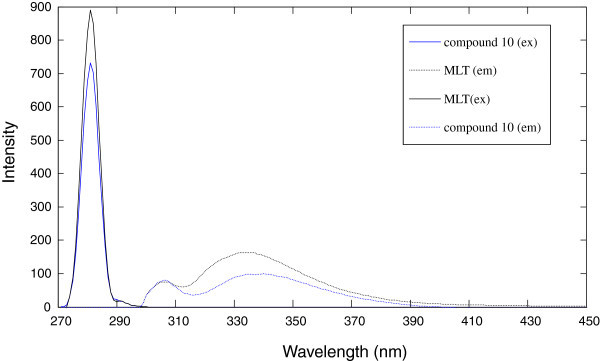
**Zero order excitation and emission spectra of 40 ng ml** ^**-1**^ **of MLT and compound 10 in methanol.**

Spectrofluorometric technique affords a higher sensitivity when compared with chromatographic ones. Both MLT and compound **10** exhibited native fluorescence in methanol with λ emission of 333 after excitation at 279 nm showing great similarity in their emission spectra. This fact hindered the direct determination of MLT in the presence of compound **10** (Figure 2).

In order to ascertain whether determination of MLT in the presence of compound **10** was feasible, the influence of the variables potentially affecting the fluorescence intensity or the position of the emission maxima was studied. The stability of the drug solutions was checked as a function of the preparation time and pH. The fluorescence intensity of MLT and compound **10** solutions maintained at 4°C was found not to vary within two weeks after preparation. Fluorescence intensity and fluorescence range of MLT and compound **10** were stable in pH ranges 2.8-11.2 as they are indole derivatives [22]. Also the effect of diluting solvent was checked and it was found that methanol and ethanol gave higher sensitivity than water. Fluorescence intensity for both MLT and compound **10** was stable for at least 2 h.

### Second derivative method

**Figure 3:**
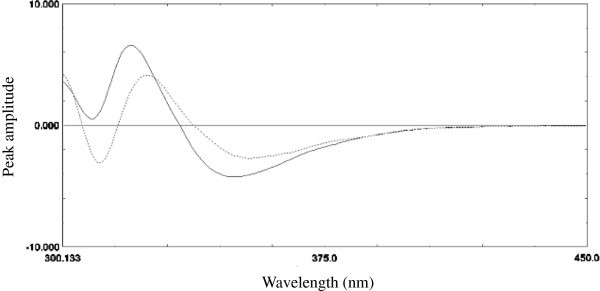
**First order derivative emission spectra of 40 ng ml** ^**-1**^ **of MLT (–) and compound 10 (…) in methanol using λ**_**excitation**_ **at 279 nm.**

**Figure 4:**
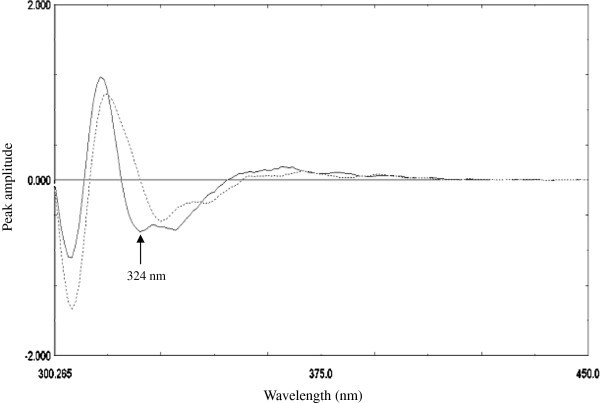
**Second order derivative emission spectra of 40 ng ml** ^**-1**^ **of MLT (–) and compound 10 (…) in methanol using λ**_**excitation**_ **at 279 nm.**

By examining first and second-order derivative curves of both MLT and compound **10** emission spectra (Δλ = 10 nm) (Figures 3 and 4), it was clear that MLT can be determined solely by measuring peak amplitude of the second-order derivative at 324.0 nm where compound **10** gave zero response (zero- crossing point). The proposed method was validated according to ICH-guidelines [23] regarding linearity, sensitivity, accuracy, specificity, repeatability and reproducibility.

#### Linearity and sensitivity

**Figure 5:**
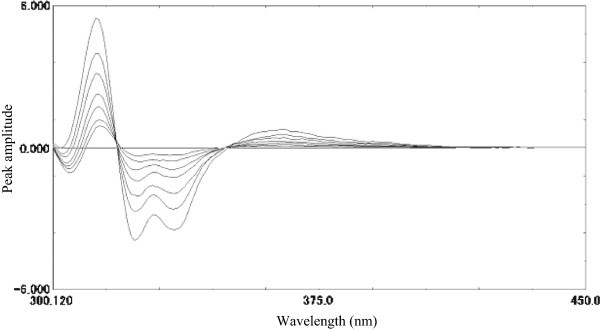
**Second order derivative emission calibration spectra of MLT in methanol (20-220 ng ml** ^**-1**^ **) using λ**_**excitation**_ **at 279 nm.**

A linear correlation was obtained between peak amplitude of second derivative spectra at 324 nm and concentrations of MLT in a range of 20–220 ng ml^-1^ (Figure 5).

The regression equation was:


Where P is the peak amplitude of the second derivative spectrum of MLT at 324.0 nm, C is the concentration of MLT in ng ml^-1^ and r is the correlation coefficient. LOD and LOQ were calculated according to the following equations [23]:


Where, σ is the standard deviation of the intercept of regression line and S is the slope of regression line of the calibration curve. All results are shown in Table 2.

**Table 2:** **Validation report of the proposed second derivative spectrofluorimetric method for determination of MLT**

Parameters	MLT
Linear range (ng ml^−1^)	20-220
Intercept ± SD	0.28 ± 0.906
Slope ± SD	1.001 ± 0.008
Correlation Coefficient	0.9995
LOD (ng ml^-1^)	2.988
LOQ (ng ml^-1^)	9.055
Accuracy^a^	100.70 ± 1.772
Repeatability^a^	99.81.32 ± 1.799
Intermediate precision^a^	100.83 ± 2.445

^a^ corresponding values are average of three determinations ± SD.

#### Accuracy

The accuracy of the proposed method was tested by analyzing triplicate samples of MLT solutions. The recovery % (mean ± SD) was 100.70 ± 1.772. These results revealed excellent accuracy (Table 2). The results obtained by applying the proposed method for determination of MLT in tablets were statistically compared with those results obtained by the reference spectrophotometric method [24]. It was concluded that with 95% confidence, there is no significant difference between them, since the calculated *t and F* values are less than the theoretical values [25] (Table 3).

**Table 3:** **Analysis of MLT in commercial tablets by the proposed and reference methods**

Method	Second derivative method	PCR method	PLS method	Reference method^24^
Mean ± SD^a^	100.51 ± 2.663	100.99 ± 1.483	100.79 ± 1.479	100.59 ± 3.379
n	5	5	5	5
*t* test (2.306)	0.041	0.247	0.124	-
F (6.388)	1.610	5.187	5.220	-

^a^ Mean ± standard deviation.

Values in parentheses are theoretical values for t and F at *P* = 0*.*05.

#### Repeatability and reproducibility

Intra-assay precision was assessed by analyzing varying concentrations of MLT (40, 60 and 80 ng ml^-1^) in triplicate in one assay batch. The inter-assay precision was assessed by analyzing the same concentrations in triplicate on 3 successive days (Table 2). The average Recovery % around 100% and low SD indicates high accuracy and high precision of the proposed method, respectively.

#### Specificity

MLT was determined in laboratory prepared mixtures containing different percentages of compound **10.** The recovery % (mean ± SD) of 101.09 ± 1.701 proved the high specificity of the proposed method for quantifying MLT in presence up to 60% of compound **10** (Table 4). Specificity was also investigated by observing any possible interferences from excepients in commercial MLT tablets, such as talc, magnesium stearate, dicalcium phosphate, and microcrystalline cellulose. These excipients did not interfere with the proposed method as indicated from the obtained good recovery values for the analysis of commercial MLT tablets (Table 3).

**Table 4:** **Determination of MLT in laboratory prepared mixtures containing different percentages of compound 10 using the proposed methods**

Mix. No.	% of compound 10	Concentration of MLT(ng ml^-1^)	Second derivative method	PCR method	PLS method
			Recovery % of MLT	Recovery % of MLT	Recovery % of MLT
1	7	80	101.11	100.45	100.15
2	20	80	102.69	101.72	101.43
3	40	60	99.75	100.06	99.77
4	50	100	102.84	100.62	100.32
5	60	40	99.05	101.57	101.28
			101.09 ± 1.701	100.88 ± 0725	100.59 ± 0.728

### PCR and PLS chemometric methods

Two chemometric methods – PCR and PLS – were applied for the determination of MLT in the presence of compound **10**. PCR and PLS methods involve the decomposition of the experimental data, such as spectrofluorimetric data in this case, into systematic variations (principal components or factors) that explain the observed variance in data. The purpose of both methods is to build a calibration model between the concentration of the analyte under study (MLT in our case) and the factors of the data matrix. The main difference between PLS and PCR methods is in the process of the decomposition of the experimental data. PCR performs the decomposition of data matrix into principal component without using the information about the analyte concentration. On the other hand, PLS performs the decomposition using both spectrum data matrix and analyte concentration [16]. The first step in the determination of MLT in presence of compound **10** by PCR and PLS methods, involves constructing the calibration matrix for the binary mixture. In this study calibration set was optimized with the aid of the multilevel multifacor design method [15]. Table 1 shows the composition of the 16 calibration samples. The emission spectra of these mixtures were collected and examined, the near zero fluorescence intensity after 380 nm accounted for the rejection of this part from the spectra. The selection of the optimum number of factors for the PCR and PLS methods was a very important pre-construction step: if the number of factors retained was more than required, more noise would be added to the data; if the number retained was too small, meaningful data that could be necessary for the calibration might be discarded. In this study, the leave-one out cross-validation method was used and the RMSECV values of different developed models were compared. Two factors were found suitable for both PCR and PLS methods. To validate the predictive ability of the suggested models, PCR and PLS methods were employed to predict the concentration of MLT in five laboratory-prepared mixtures (validation samples) containing different percentages of compound **10**, where satisfactory results were obtained (Table 4). The predicted concentrations of the validation samples were plotted against the known concentrations to determine whether the model accounted for the concentration variation in the validation set. Plots were expected to fall on a straight line with a slope of 1 and zero intercept. MLT, in all samples, lay on a straight line and the equations of these lines were y = 0.994 x − 0.205 (r = 0.9995) for PCR and y = 0.997 x − 0.205 (r = 0.9995) for PLS. Both plots had a slope of almost 1 and an intercept close to zero. The proposed PCR and PLS methods were successfully used for the determination of MLT in commercial preparations (Table 3). The results obtained by applying PCR and PLS methods for determination of MLT in tablets were statistically compared with those results obtained by the reference spectrophotometric method [24]. It was concluded that with 95% confidence, there is no significant difference between them, since the calculated *t and F* values are less than the theoretical values [25] (Table 3).

## Conclusion

In this study, simple and sensitive spectrofluorimetric methods were developed for the analysis of MLT in the presence of its main contaminant, compound **10**. Reviewing the literature exposed that there are no reports for such analysis except a sophisticated HPLC/Ms/Ms method. Accordingly, compound **10** was synthesized in adequate quantities starting from 5-methoxyindole-2-carboxylic acid (**1**) to be used for the development of appropriate spectrofluorimetric methods for routine analysis of MLT in commercial preparations. The proposed spectrofluorimetric methods combine the rapidness and simplicity advantages of traditional spectrometric methods together with other important analytical merits, such as sensitivity and specificity. Moreover, simplicity was illustrated by the minimum requirement of chemicals and solvents since methanol was the only organic solvent used in the procedure. The suggested methods were validated and can be applied for routine quality control analysis of MLT commercial tablets without prior separation or interference from impurities/excipients.
