# Erratum to: New spectrofluorimetric methods for determination of melatonin in the presence of *N*-{2-[1-({3-[2-(acetylamino)ethyl]-5-methoxy-1*H*-indol-2-yl}methyl)-5-methoxy-1*H*-indol-3-yl]-ethyl}acetamide: a contaminant in commercial melatonin preparations

**DOI:** 10.1186/s13065-016-0203-y

**Published:** 2016-09-28

**Authors:** Hany W. Darwish, Mohamed I. Attia, Darius P. Zlotos

**Affiliations:** 1 Department of Pharmaceutical Chemistry, College of Pharmacy, King Saud University, P.O. Box 2457, Riyadh, 11451 Saudi Arabia; 2 Department of Analytical Chemistry, Faculty of Pharmacy, Cairo University, Kasr El-Aini Street, Cairo, 11562 Egypt; 3 Department of Pharmaceutical Chemistry, Institute of Pharmacy and Food Chemistry, Würzburg University, Am Hubland, 97074 Würzburg, Germany; 4 Department of Pharmaceutical Chemistry, Faculty of Pharmacy and Biotechnology, The German University in Cairo, New Cairo City, Egypt

## Erratum to: Chemistry Central Journal 2012, 6:36 DOI 10.1186/1752-153X-6-36

In the original article [1] Professor Darius Zlotos was listed in the acknowledgments section. We have now been informed by the Standing Commission for the Investigation of Scientific Misconduct of the University of Wurzburg that he should be included as an author.

We would also like to correct two typographical errors in the original article [1]:

- in the Background section ‘oesinophilia-myalgia’ should be ‘eosinophilia-myalgia’,
- in the Experimental section ‘Merc Inc’ should be ‘Merck Inc’.

We regret any inconvenience caused by these errors.
